# Production Characteristics and Management Practices of Indigenous Tswana Sheep in Southern Districts of Botswana

**DOI:** 10.3390/ani12070830

**Published:** 2022-03-25

**Authors:** Monosi Andries Bolowe, Ketshephaone Thutwa, Phetogo Ineeleng Monau, Cosmas Malejane, Patrick Monametsi Kgwatalala

**Affiliations:** Department of Animal Sciences, Botswana University of Agriculture and Natural Resources, Private Bag, Gaborone 0027, Botswana; mabolowe@yahoo.com (M.A.B.); pmonau@buan.ac.bw (P.I.M.); cmalejan@buan.ac.bw (C.M.); pkgwatal@buan.ac.bw (P.M.K.)

**Keywords:** breeding practices, husbandry, traits preferences, Tswana sheep

## Abstract

**Simple Summary:**

Indigenous Tswana sheep are an important agricultural resource that largely contribute to the economic, socio-cultural and spiritual aspects of resource-poor farmers. Therefore, as was the aim of this study, exploring and describing the production system in which they are raised, their management including breeding practices is thus crucial to inform researchers, farmers, and policymakers on the formulation of sustainable improvement strategies, sustainable utilization and conservation of these vital animal genetic resources. In the southern part of Botswana, indigenous Tswana sheep are mostly kept by single males aged 51–60 mostly with primary and secondary levels of education. Most Tswana sheep are kept for cash generation through sales, followed by meat consumption and ceremonial obligations, especially in the southern district. Most farmers across districts castrate their young rams at over 3 months of age and most farmers rely on natural pastures for feeding. Feed supplementation is conducted mostly during the dry season while no supplementation is generally practiced during the wet season as there is plenty of palatable forage. Most farmers prefer Tswana rams over exotic breeds because of their ability to survive and reproduce under the Botswana harsh environment. This information is crucial in the formulation of sustainable improvement strategies.

**Abstract:**

The aim of this study was to describe the indigenous Tswana sheep production systems, their management and farmers’ preferred selection traits when selecting breeding rams in four southern districts of Botswana. A total of 105 households; Kgatleng (*n* = 30), Kweneng (*n* = 27), southern (*n* = 24) and south–east (*n* = 24) districts were interviewed using structured questionnaire. An index-based approach was used to rank farmers’ most preferred traits for their production systems. Data were analyzed using Statistical Package for Social Sciences. The Chi-square test was used to assess the statistical significance among categorical variables. The results indicated that indigenous Tswana sheep are mainly kept by males, single people, aged between 51 and 60 years possessing primary and secondary education. Management practices across the districts include castration, health care and supplementation mostly during the dry season. Superior fitness traits of indigenous Tswana rams over exotic rams were considered more important when selecting breeding rams in Kgatleng, Kweneng and south–east while in the southern district, rams were mainly selected based on body size. Most farmers kept breeding rams while those who did not keep rams depended on communal rams for service. This information is important in designing successful breeding programs and strategies for the conservation and sustainable utilization of indigenous Tswana sheep genetic resources.

## 1. Introduction

Sheep play an important role in the socio-economic lives of people around the world including in Botswana [1]. They are kept for their economic and sociocultural value [2], [3] to mankind as well as agricultural productivity for the present and posterity [4]. They provide their owners with a wide variety of products and services such as meat, milk and immediate cash income [5]. Compared to large ruminants such as cattle, sheep are highly prolific, have shorter generation intervals and generally require low capital investment [6]. In addition, sheep require small space and feed hence ideal to be kept by resource-poor smallholders, especially in areas where there is minimal grazing land.

**Figure 1:**
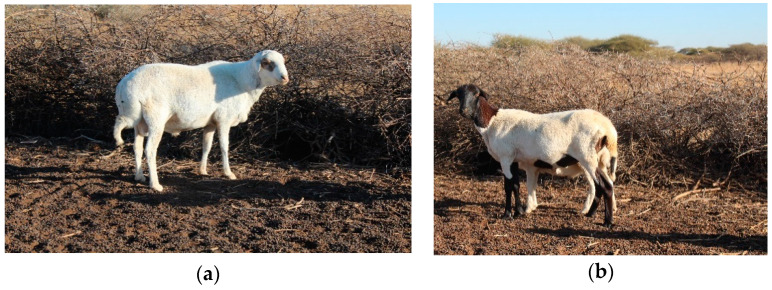
(**a**) Indigenous Tswana ram, (**b**) Indigenous Tswana ewe: Pictures of indigenous Tswana sheep.

In Botswana, there are about 300,000 sheep of various breeds of which 65% are indigenous Tswana sheep [7]. The indigenous Tswana sheep (Figure 1) dominate the national flock population. They are mainly bred for meat production, and it is uncommon for them to be milked. Indigenous Tswana sheep are highly adapted to prevalent harsh environmental conditions and resilience to endemic diseases. They can walk long distances and survive well on low-quality forages [1]. Under such environmental conditions, the performance of Tswana sheep with regards to survival and production is better than its exotic counterparts such as Dorper [8]. Despite all these, the existence of Tswana sheep is under threat of immensely being replaced by the less adapted exotic breeds and crossbreds [7] that are of high maintenance costs to resource-poor rural farmers. This is because farmers practice uncontrolled crossbreeding that is performed without evaluating and setting optimum breeding goals [9].

There is a need to develop realistic breed improvement programs that will ensure sustainable utilization of local animal genetic resources (AnGR). The prerequisite to achieving this is to first understand their existing natural production environment and the current context of their utilization. This includes indigenous knowledge on selection, management and identification of breeding goals [10]. In Botswana, there is a paucity of such information. There is no clearly defined breed documentation or performance evaluation so that the breed is kept mostly under subsistence production systems where breeding records are not kept. Previous studies that described the production systems of indigenous Tswana sheep are not only scarce and limited in their scope to provide an in-depth analysis of the sheep production systems. The aim of this study was therefore to characterize existing indigenous Tswana sheep production systems, investigate the management practices practiced by rural farmers keeping indigenous Tswana sheep and establish farmers’ preferred traits in the selection of breeding rams in the southern part of Botswana. This information will be useful in guiding policymakers in devising strategies to improve productivity and sustainable utilization of Tswana sheep and inform conservation programmes of the breed.

## 2. Materials and Methods

### 2.1. The Sampling Procedure

A multi-stage purposive sampling technique was employed for the selection of districts for the study. In the first stage, discussions were held with district agricultural experts of the Department of Veterinary Services to know the distribution of indigenous Tswana sheep population in each district in Botswana. Based on the distribution, four districts (Kgatleng, Kweneng, south–east and southern) were selected for the survey study. Random sampling was then used to select representative villages within districts and households/farms within villages. In each randomly selected village, four to five households were randomly selected for the survey study. Individual farm/household visits were made by the team which comprised extension area veterinary workers and researchers who briefed households about the objectives of the study and administered the questionnaire after consent was given.

### 2.2. Data Collection

**Figure 2:**
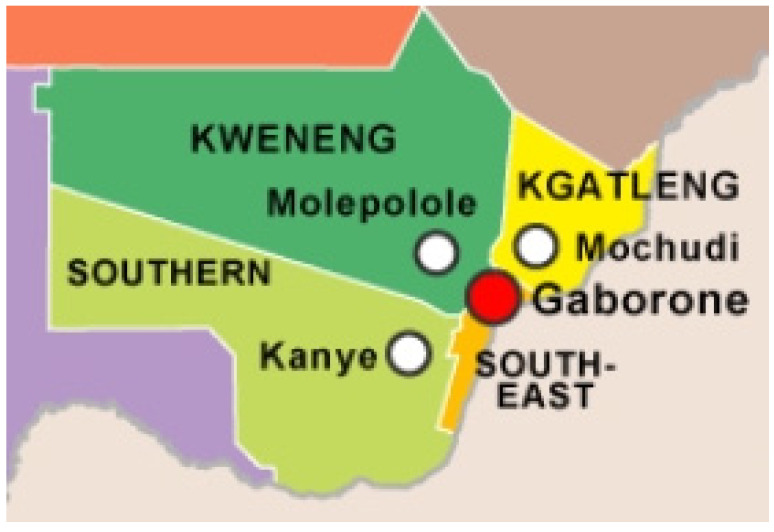
Map showing geographical location of Districts selected for the study (http://mapsopensource.com/botswana-districts-map.html, accessed on 21 March 2021).

A survey was conducted in Kgatleng (24°15′ S 26°30′ E), Kweneng (24°00′ S 25°00′ E) south–east (25°00′ S 25°45′ E) and southern (25°00′ S 25°00′ E) districts of Botswana (Figure 2) from November 2020 to January 2021. A structured questionnaire and visual observations were used to investigate and collect information on the production and management systems applied to indigenous Tswana sheep from a total of 105 households in the four districts of southern Botswana namely; Kgatleng (*n* = 30), Kweneng (*n* = 27), southern (*n* = 24) and south–east (*n* = 24). The questionnaire was a slightly modified version of those designed and recommended for livestock breed surveys in Africa [11,12]. The questionnaire included socio-economic parameters (demographic) (e.g., gender, age, marital status and education level of indigenous Tswana sheep farmers), breeding management and methods of watering sheep, feeds and feeding management of the sheep, selection criteria and major production systems of each respondent in each district. Participants were asked to rank their major sources of income, to describe their ram selection criteria (ranging from1 to 3, where 1 = most important and 3 = least important). Information was obtained through an oral interview for which individual consent was given by the farmers.

### 2.3. Data Analysis

The data from the questionnaires were coded, entered and analyzed using the Statistical Package for Social Sciences (SPSS, 2007). The descriptive statistics, frequency and cross-tabulation procedures were used to analyze the data. Chi-square test (X^2^) was used to assess the statistical significance at *p* ≤ 0.05 level of significance.

### 2.4. Index-Based Ranking

Individual farmers ranked their criteria for preferred traits in ram selection and a weighted ranking approach was adopted to determine the relative importance of each criterion to a household. This approach was used for ranking major sources of income, reasons for keeping sheep and the criterion used in the selection of rams. The formulas by [13,14] were adopted for the weighted criteria as follows:

(1) $$\mathrm{Relative}\;\mathrm{importance}\;\mathrm{index}=\frac{\sum w}{AN}\;=\frac{3n_{3}+2n_{2}+1n_{1}}{3N}$$

where *w* is the weighting given to each factor by the respondent, ranging from 1 to 3. For example, *n*_1_ = number of respondents for least important, *n*_2_ = number of respondents for fairly important and *n*_3_ = number of respondents for most important. *A* is the highest weight (i.e., 3 in this study) *N* is the total number of respondents. The relative importance index ranges from 0 to 1 [13,14].

## 3. Results

This section may be divided into subheadings. It should provide a concise and precise description of the experimental results, their interpretation, as well as the experimental conclusions that can be drawn.

### 3.1. Household Characteristics

The proportion of male-headed households was higher than the proportion of female-headed households (77.78% vs. 22.22%). A large proportion of respondents (41.9%) were aged between 51 to 60 years (Table 1). A higher proportion of respondents from Kgatleng (67.7%) and south–east (54.2%) were not married while an equal proportion of married to unmarried respondents was observed in Kweneng (51.9%:48.1%) and southern (50%:50%) districts. A high proportion of the head of households in Kgatleng (40.0%), Kweneng (40.7%) and south–east (54.2%) attained primary education and 62.5% of southern district heads of households attained secondary education. Respondents in all regions were small-scale communal farmers practicing mixed crop-livestock farming. The livelihood of most farmers (79%) was based on livestock production and those farmers raised their sheep under an extensive production system in communal areas.

### 3.2. Livestock Holding per Household and Flock Structure

Most of the livestock species kept in the districts include cattle, sheep, goats, poultry, donkeys and pigs. There were no significant differences in the number of sheep per household across the four districts although the southern district had more sheep per household (30.08 ± 3.77) (Table 2).

### 3.3. Purpose of Keeping Sheep

The reasons for which sheep are reared by the agrarian society in the study areas are presented in Table 3. The primary purpose of keeping sheep in Kgatleng and Kweneng districts is for generating additional sources of income from animal sales (index = 0.480 and 0.390, respectively) followed by investment (index = 0.231 and 0.221, respectively) and meat consumption (index = 0.211 and 0.210, respectively). Southern and south–east district farmers primarily kept sheep for ceremonies (socio-cultural) (index = 0.310 and 0.371, respectively) followed by cash sales and meat consumption. The major source of income for Kweneng (0.513) and southern (0.501) districts farmers was livestock and livestock products whilst wages/salaries were the major source of income for Kgatleng (0.546) and south–east (0.570) farmers.

### 3.4. Breeding Management and Selection of Ram Genotypes

The majority of farmers across study districts (98.1%) practiced uncontrolled mating (Table 4). More farmers (81.9%) kept rams for breeding purposes whilst 18.1% did not keep rams and depended on communal rams for service. Farmers in Kgatleng (46.7%), Kweneng (77.8%) and southern districts (75%) bred with their own rams originating from their own flock whilst South-East farmers (33%) predominantly used donated rams. The Tswana breed rams were the main breeding rams used in Kgatleng, Kweneng and Southern districts whilst south–east district farmers preferred the Tswana and exotic crossbred genotypes (33%) and a combination of Tswana rams alongside crossbreds (29.2%).

### 3.5. Farmers Preferred Trait Ranking

Selection of parents of the next generation is predominantly performed in rams across the sampled farmers and all mature females are usually allowed to parent the subsequent generation. Competitive performance of indigenous rams in Kgatleng (0.290), Kweneng (0.301) and south–east (0.247) was considered the most important trait, followed by body size in Kgatleng (0.251), Kweneng (0.262) and south–east (0.244) (Table 5). In the southern district, rams were selected primarily for body size (0.372) and body conformation (0.311).

### 3.6. Castration Practice

The reason for castration reported by most farmers across the four districts (99%) was primarily to control breeding (77.1%) followed distantly by temperament improvement plus controlling breeding (14.3%) and to a lesser extent controlling breeding plus to improve meat quality (7.6%). Farmers in the Kgatleng (50%), Kweneng (48.1%) and south–east (58.1%) districts castrate their rams at 3–6 months of age whilst southern district (50%) castrate their rams at 6–12 months.

### 3.7. Feeding and Supplementation

The results indicating the feed resources for indigenous Tswana sheep during both the dry and wet seasons are presented in Table 6. The results indicate that there were hardly any differences between the four districts in terms of feeding regime in both seasons. Most of the respondents across districts solely depended on freely grazing their sheep on natural pasture in communal grazing land as the major feed source during both the dry and wet seasons. Overall, 53.3% of farmers across districts supplemented with roughages/crop residues during the dry season while a small fraction (12.9%) of farmers across districts supplemented with crop residues and bought in concentrates. Supplementation with salt mineral licks and vitamins was not common across the study districts. On the other hand, 64.8% of farmers across the four districts did not supplement during the wet season.

### 3.8. Diseases and Health Management

**Figure 3:**
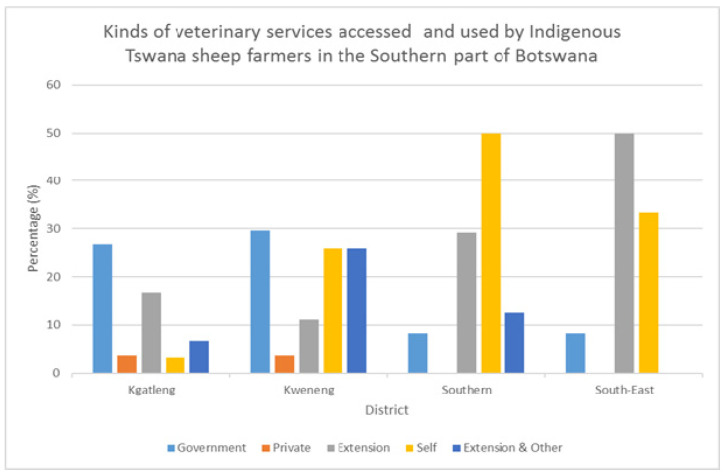
Veterinary services accessed and used by Indigenous Tswana sheep farmers in the four districts of Botswana.

Less or no disease load was reported across districts except occasional pasteurellosis. Parasitic diseases, particularly heart water, were reported in lesser incidences in Kweneng district, especially after the rainy season. However, typical disease symptoms such as coughing, sneezing, bloating and substantial mucus discharged from the nostrils were common across districts. The majority of the farmers had access and used modern medication and hardly used ethno-veterinary practices for disease control. The modes of accessing veterinary services across districts are summarized in Figure 3.

## 4. Discussion

There is an urgent need to unravel and understand the characteristics of the production environment and breeding practices underlying the production of indigenous Tswana sheep in order to design holistic and sustainable breed improvement programs.

### 4.1. Household Characteristics

The high proportion of non-married people keeping sheep in Kgatleng might be due to social reasons because it is easier to resort to small stock farming to maintain the standard of their lives than going for cattle farming. More farmers in Kgatleng, Kweneng and south–east districts with primary education is consistent with [15] who reported that most sheep farmers from Adiyo kaka (70.2%) and Horro (48.7%) regions of Ethiopia attained primary school level of education. The literacy level of the farmers provides them with an opportunity to devise informed interventions in response to evolving production systems and enhance their management ability of the animals [10]. The low participation of youth in sheep production observed in this study is consistent with several studies that reported low participation of Botswana youth in cattle [16] and goat production [17]. This might be because youth do not see agriculture as a viable sector of employment [18] and youth could be discouraged by associated constraints such as lack of land, water, regulatory policies and financial support. This is worrisome as youth should be drivers of the country’s livestock industry for sustainable development and growth of different livestock sectors. According to [1] youth are more learned and skilled and thus it would be much easier for them to adopt modern farming technologies for enhanced agricultural production which would alleviate poverty and improve livelihoods in the rural populace.

### 4.2. Livestock Holding per Household and Flock Structure

The similar average number of sheep per household across the districts attests to the increasing popularity and importance attached to sheep across the country. Respondents in the current study preferred keeping sheep than cattle because they are resilient to diseases commonly found in the southern region and can survive on marginal feeds sourced along farm boundaries, along streams/rivers and roadsides compared to cattle. Similar reasons for preference of sheep over cattle have been reported by [19] in Alaba Special Woreda farmers in Southern Ethiopia.

### 4.3. Purpose of Keeping Sheep

Understanding the purposes for which farmers keep sheep is important in formulating breeding goals in the tropics [20] and should not be ignored if breed improvement and conservation programmes are to be drawn. The multifaceted roles played by sheep in the livelihoods of farmers identified in this study are a direct reflection of the farmers’ multiple objectives for sheep production. The preference of sheep in generating household income via sales reported in Kgatleng and Kweneng districts is consistent with [20] who outlined the importance of livestock in generating income for small ruminant farmers in Kenya amongst other purposes. The southern and south–east district farmers prioritized non-tangible benefits of keeping sheep-like ceremonial value over the generation of income. According to farmers in the south–east and southern districts, the esteemed preference and use of sheep in ceremonies particularly weddings is symbolic in that sheep are considered naturally noble animals that brides should emulate in their behavior towards their in-laws.

The high dependency of Kgatleng and south–east farmers on wages and salaries as a major source of income observed in this study was attributed to their proximity to urban or peri-urban areas where they have other means of cash flow income other than agriculture. Furthermore, the majority of farmers in these districts are well educated and therefore have formal employment hence wages/salaries as their main source of income. A similar observation was made by [17] who reported that indigenous Tswana goat farmers in the southern region of Botswana preferred piece jobs as a major source of income over livestock sales and that was attributed to their proximity to urban or peri-urban areas.

### 4.4. Breeding Management and Selection of Ram Genotypes

The observed uncontrolled mating across districts was associated with a high dependency of farmers on grazing in unfenced communal grazing lands. Furthermore, [15] also reported a similar practice for Adiyo kaka and Horro sheep in Ethiopia. Since most farmers owned their own breeding rams, it may imply that animals within a flock are closely related thus chances of inbreeding are high. Low entry of males into the flocks either through purchase or other means may further escalate the inbreeding levels, especially in small-sized flocks [15]. Therefore, preventing uncontrolled mating in communal grazing areas could be a way to minimize the deleterious effects of inbreeding, especially when coupled with other practices such as castration of males at an early age and rotational use of breeding males. There are no formal community-based breeding programmes designed by authorities for smallholders in Botswana to help with breeding issues. However, farmers in the study sites are gradually devising and adopting newer plans and farming methods to collectively combat the effects of uncontrolled mating. For example, their breeding rams remain in the ploughing fields while females are allowed to graze in communal areas alone as a way of controlling mating to further reduce inbreeding.

The majority of farmers reported that they recognize the importance of the selection of rams and practiced it to some extent using their own criteria. The majority of the farmers in Kgatleng, Kweneng and southern districts chose and preferred own-bred Tswana rams for breeding because though not formally recorded, the majority of them voiced that they considered indigenous rams to have better survival and reproductive performance or traits than exotic breeds under Botswana production environment. This is because they believe that survival is more important than fast growth and good appearance, hence indigenous genotypes are given more preference when selecting breeding rams in the three aforementioned districts. However, a paradigm shift in farmers’ breeding goals towards generating income through animal sales was observed across districts but was more evident in the south–east as more farmers are beginning to prefer crossbred genotypes in mating. The farmers breeding goals are more focused on a few economically important traits of high market value such as meat proportion using exotic genotypes and their crosses. They thus practiced uncontrolled crossbreeding using Tswana-exotic crossbred rams in pursuit of taking advantage of breed complementarity [9] and heterosis effects [21]. This is conducted to meet the urban market demand for mutton in the capital city of Gaborone which is proximate to the districts. Ultimately, this will increase income but unfortunately might influence adaptation, lower immunity, increase the susceptibility of the resultant crossbreds to diseases and may lead to the subsequent replacement of the once locally adapted breeds by the high-yielding international or trans-boundary breeds.

### 4.5. Farmer Preferred Trait Ranking

The use of preferences based on indices is a powerful tool for farmers to objectively and accurately rank their animals [22]. Adaptation traits (disease tolerance/resistance, drought tolerance) summed up as superiority in competitive performance of Tswana sheep over its exotic counterparts under tropical conditions was considered the most important trait by Kgatleng, Kweneng and south–east district farmers. The present study recognizes that adaptation traits are equally or more important than production traits to these farmers. Although highly adapted animals were desired, farmers also underscored the need for income from the sale of animals hence a substantial proportion of farmers also ranked body size as a valuable trait of economic importance for them. The preference of production traits (body size) by southern district farmers is attributed to their primary purpose for keeping sheep (ceremonial purposes where large-bodied animals are preferred) hence the need for well grown, structurally sound and large-bodied animals. The desire for larger animals that can catch a better selling price/income has also been reported for farmers in Kenya [23].

### 4.6. Castration Practice

Castration for the purposes of controlling unwanted mating has been reported in the literature [24,25]. Although it is commonly recommended that castration should be performed at an earlier age in weeks, farmers in the study generally castrated their rams at a later age of more than 3 months, especially southern district farmers. This finding is consistent with the report of [15] who reported an average castration age of 10.8 ± 2.5 months in Adiyo Kaka rams and [24] who reported an average castration age of 12.09 ± 4.10 months in Doyogena rams. This is because some sheep producers believe that castration at an early age ‘stunts growth’. Entire males produce hormones that enhance faster growth rate than castrates, therefore castrating at 6–12 months of age gives the farmer some of the benefit of the increased male hormone growth effect before the animals are castrated. This is supported by the trials of [26] who reported that carcasses of entire Awassi male lambs were up to 1 kg heavier than those of Awassi castrates of the same age and they also had a lower fat content and thus improved meat quality.

### 4.7. Feeding and Supplementation

Inadequate feeding and poor-quality feed especially during the dry season are often regarded as major factors limiting sheep production [20]. The dependency of farmers on communal areas to meet the nutritional requirements of their animals as revealed in the current study concurs with the findings of [3,27]. In addition to natural pasture, some farmers across districts practiced supplementary feeding during both the dry and rainy seasons using a variety of feed resources including roughages/crop residues, mineral blocks and bought concentrates. This supplementary feeding is ascribed to unreliability and poor nutritive value of the scarce roughage, especially during drought/dry periods. The low proportion of farmers who use salts and vitamins as supplements for sheep in the current study is inconsistent with [15] for Horro and Adiyo Kaka sheep and [28] for Gumuz sheep in Ethiopia. Lack of supplementation during the wet season as reported in this study is attributed to an abundance of natural pasture in both quantity and quality. Furthermore, during the survey, it was observed that as a general management practice, sheep were kept for longer hours in kraals during the wet season than the dry season. The late release of sheep for grazing during the wet season might be attributed to the fact that animals graze to their satiety in shorter grazing time due to pasture availability. A similar observation was reported by [3] for KwaZulu-Natal sheep in South Africa. In further agreement with this observation, [15] reported that sheep in Adiyo Kaka grazed for a shorter time (7.4 h a day) during the wet season and grazed for a longer time (9.6 h a day) during the dry season as a strategy to cope with a feed shortage.

### 4.8. Diseases and Health Management

The common symptom of the mucus discharged from sheep nostrils was probably due to lack of vaccination against internal parasites, pasteurella and pulpy kidney. *Pasteurella multicoda*, one of the pasteurellosis-causing pathogens, causes nasal discharge in sheep [29]. Disease symptoms incidences and transmission were common as most sheep flocks freely roam and mix with each other at communal grazing areas and at shared drinking points. Similar findings have been previously reported by [19] for Alaba sheep of Ethiopia. The low proportion of farmers using ethno-veterinary services across districts could be because most of the farmers received formal education and may be less knowledgeable about ethno-veterinary practices hence resorting to modernized herd health practices. These findings concur with [3] who reported that a few farmers in their study used traditional plants as supplements and for veterinary use. Contrary to the current study, [19] reported that 90% of participants in their study in Ethiopia used traditional medication and the remaining 10% used modern veterinary drugs.

## 5. Conclusions

Indigenous Tswana sheep are kept in a mixed-crop-livestock system in the communal areas of the southern part of Botswana. Sheep serve socio-economic and cultural values apart from providing meat, milk and non-profitable benefits such as manure. Indigenous Tswana sheep are mainly kept by males, single people, aged between 51 and 60 and possessing primary and secondary levels of education. Most farmers depend on free grazing as the major feed source with supplementary feeding practiced only during the dry season when feed quality and quantity are compromised. Performance, in terms of survival and reproductive ability, body size and conformation were the highly preferred traits in selecting rams. Farmers prefer keeping Tswana rams originating from their own flocks for breeding purposes and prefer castration of males to be performed at a later stage of 6–12 months.

## Figures and Tables

**Table 1:** Socio-economic characteristics of households (%) keeping sheep in the four regions of Botswana.

Descriptors	Kgatleng (%)	Kweneng (%)	Southern (%)	South-East (%)	Overall Total (%)	X^2^ *p* Value
Gender						
Male	83.3	77.8	83.3	66.7	77.78	
Female	16.7	22.2	16.7	33.3	22.22	0.44
Age (years)						
≤30 years	3.3	0	0	0	0.83	
31–40	16.7	0	25.0	16.7	14.6	
41–50	20.0	29.6	20.8	20.8	22.8	
51–60	43.3	40.7	33.3	50.0	41.83	0.35
61–70	13.3	18.5	20.8	12.5	16.3	
>70	3.3	3	0	0	1.6	
Not known	0	0	0	0	0	
Marital status						
Married	33.3	51.9	50	45.8	45.25	
Single	67.7	48.1	50	54.2	55.00	0.42
Level of Education						
None	0	3.7	12.5	0	4.05	
Primary	40.0	40.7	16.7	54.2	37.9	0.10
Secondary	36.7	37.0	62.5	12.5	37.18	
Tertiary	23.3	18.5	8.3	33.3	20.85	
Is Livestock major activity						
Yes	93.3	96.3	62.5	58.3	79	0.01
No	6.7	3.7	37.5	41.7	21	
Type of production system						
Intensive/industrial	0	0	4.2	0	1.05	
Extensive/pastoral	86.7	96.3	91.7	75.0	87.43	0.10
Semi intensive	13.3	3.7	4.2	25.0	11.55	

**Table 2:** Average number of livestock species per household in the four surveyed districts of Southern Botswana.

Species	Kgatleng	Kweneng	Southern	South-East
Sheep	22.20 ± 3.8	24.81 ± 3.56	30.08 ± 3.77	23.58 ± 3.77
Goats	25.633 ± 5.0	32.56 ± 5.25	35.54 ± 5.57	24.29 ± 5.57
Cattle	13.03 ± 2.84 ^ab^	17.96 ± 2.99 ^a^	5.75 ± 3.17 ^bc^	4.04 ± 3.17 ^c^
Chicken	11.20 ± 1.79 ^a^	8.74 ± 1.89 ^a^	2.75 ± 2.00 ^b^	9.42 ± 2.00 ^a^
Donkeys	2.23 ± 0.43 ^ab^	2.23 ± 0.46 ^a^	0.17 ± 0.48 ^bc^	0.96 ± 0.48 ^c^
Pigs	0.27 ± 0.13	0.15 ± 0.15	0.13 ± 0.15	0.04 ± 0.15

^a,b,c^ Means across rows with different superscripts were significantly different at (*p* < 0.05).

**Table 3:** Indices and their ranking for reasons for keeping sheep and source of income by respondents in surveyed districts of Botswana.

Descriptors	Kgatleng Index	Rank	Kweneng Index	Rank	Southern Index	Rank	South-East Index	Rank
Purpose of Keeping Sheep								
Meat	0.211	3	0.210	3	0.180	4	0.160	4
Investment	0.231	2	0.221	2	0.211	3	0.250	2
Ceremonies	0.030	4	0.171	4	0.310	1	0.371	1
Cash	0.480	1	0.390	1	0.231	2	0.201	3
Dowry payment	0.031	4	0.000	5	0.000	5	0.000	5
Cultural rites	0.020	5	0.000	5	0.000	5	0.000	5
Source of Income								
Crop production	0.130	3	0.208	3	0.300	2	0.191	3
Livestock and products	0.324	2	0.513	1	0.501	1	0.230	2
Wages/salaries	0.546	1	0.279	2	0.181	3	0.570	1

**Table 4:** Frequency of mating systems, source and breeds of breeding rams in the four surveyed districts.

Descriptors	Kgatleng (%)	Kweneng (%)	Southern (%)	South-East (%)	Overall (%)
Mating					
Uncontrolled	93.3	100	100	100	98.1
Controlled	6.7	0	0	0	1.9
Source of breeding ram					
Own ram (self-bred)	46.7	77.8	75	12.5	53
Own ram (bought)	10	0	0	16.7	6.68
Donated ram	6.7	0	12.5	33.3	13.13
Communal area ram	23.3	11.1	12.5	16.7	15.9
Own ram (self-bred) and own ram (bought)	13.3	7.4	0	20.8	10.38
Borrowed ram	0	3.7	0	0	0.9
Breed of ram					
Indigenous Tswana	26.7	44.4	58.3	16.7	36.5
Pure exotic (Dorper)	10	11.1	4.2	0	6.3
Indigenous x exotic cross	23.3	14.8	12.5	33.3	20.98
Tswana and Dorper	3.3	0	0	0	0.8
Tswana, Dorper and cross	3.3	0	0	0	0.8
Tswana and cross	3.3	0	4.2	29.2	9.2
Dorper and cross	6.7	0	8.3	0	3.75
Tswana, cross and beef master	0	0	0	4.2	1.05
No ram	23.3	11.1	12.5	16.7	15.9

**Table 5:** Ranking of selection criteria of rams in the four surveyed Districts of Botswana.

Characteristic	Kgatleng Index	Rank	Kweneng Index	Rank	Southern Index	Rank	South-East Index	Rank
Body size	0.25	2	0.262	2	0.372	1	0.244	2
Body conformation	0.147	4	0.212	3	0.311	2	0.198	4
Temperament	0.17	3	0.189	4	0.207	3	0.213	3
Performance	0.29	1	0.300	1	0.068	4	0.247	1
Availability	0.083	5	0.037	5	0.042	5	0.071	5

**Table 6:** Frequency (%) of supplementary regime used on sheep during the wet and dry seasons.

Supplement	Kgatleng	Kweneng	Southern	South East	Overall
Dry season					
Roughages/crop residue	46.7	66.7	50	50	53.3
Bought feed/concentrates	16.7	3.7	4.2	4.2	7.6
None	26.7	3.7	12.5	20.8	16.2
Roughages/crop residues and Mineral (Salts)/Vitamins	3.3	7.4	16.7	-	6.8
Roughages/crop residue and bought feed/concentrates	3.3	14.8	16.7	16.7	12.9
Minerals (salts)/vitamins and bought feed/concentrates	3.3	3.7	-	8.3	3.8
Wet season					
Roughages/crop residue	16.7	33.3	12.5	25	21.9
Bought feed/concentrates	3.3	3.7	4.2	8.3	4.8
None	76.7	55.6	66.7	58.3	64.8
Roughages/crop residues Mineral (Salts)/Vitamins	3.3	3.7	12.5	8.3	6.95
Roughages/crop residue and feed/concentrates	-	3.7	4.2	-	1.98

## Data Availability

The data are contained within the article.
